# Genome-Wide DNA methylation profiling of tissue-plasma pairs identifies Novel cfDNA biomarkers for the diagnosis of gastric cancer

**DOI:** 10.1371/journal.pgen.1012322

**Published:** 2026-10-15

**Authors:** Hailing Ou, Jinli Miao, Fusheng Ling, Jie Gao, Tingan Wang, Zhihua Ye, Chunbo Zhang, Mingwei Huang, Yuzhou Qin

**Affiliations:** 1 The First Affiliated Hospital of Guangxi University of Chinese Medicine, Nanning, Guangxi, China; 2 The Yangtze River Delta Biological Medicine Research and Development Center of Zhejiang Province, Yangtze Delta Region Institution of Tsinghua University, Hangzhou, Zhejiang, China; 3 Department of Gastrointestinal Surgery, Guangxi Medical University Cancer Hospital, Nanning, Guangxi, China; 4 State Key Laboratory of Targeting Oncology, Guangxi Medical University, Nanning, Guangxi, China; University of Southern California, UNITED STATES OF AMERICA

## Abstract

Screening for gastric cancer (GC) is critical for improving patient survival, yet current clinical methods suffer from invasiveness and limited sensitivity. The aim of this study is to develop a machine learning model for the non-invasive diagnosis of GC using the whole-genome methylation profile of circulating free DNA (cfDNA) in plasma. Plasma cfDNA from 61 patients with GC and 29 healthy controls. First, whole‑genome bisulfite sequencing (WGBS) was performed on 29 paired GC and adjacent normal tissues to identify differentially methylated regions (DMRs). These tissue DMRs were then mapped to plasma cfDNA methylation data, yielding 32 stable DMRs that changed consistently in both tissues and plasma. Using LASSO and Random Forest (RF) algorithms, eight core diagnostic markers were selected. Four machine learning algorithms (Support Vector Machine, RF, K-Nearest Neighbor, Naïve Bayes) were used to build diagnostic models. WGBS revealed global hypomethylation and CpG island hypermethylation in GC, consistent with plasma cfDNA methylation patterns. The RF model built on the eight cfDNA methylation markers (annotated to genes including ATP8A2) performed best, achieving AUC values of 0.978 and 0.938 in the training and independent test sets, respectively, with an accuracy of 88.46%, sensitivity of 94.44%, and specificity of 75.00%. After adjusting for age, the model achieved an AUC of 0.924 on the test set, still demonstrating high discriminatory performance. Functional analysis implicated these markers in pathways such as phagocytosis and axon guidance, and showed significant correlation with immune cell infiltration. Notably, these core DMRs are not covered by the probes on the methylation arrays (450K and EPIC v2), highlighting the advantage of WGBS for novel biomarker discovery. In conclusion, tissue-plasma concordance analysis identified a panel of plasma cfDNA methylation biomarkers with strong diagnostic potential. The resulting RF model provides a promising non-invasive strategy for GC screening.

## Author summary

In this study, we set out to address the lack of non‑invasive and efficient tools for the detection of gastric cancer. Cancer cells release DNA fragments with specific methylation patterns into the bloodstream. By analyzing plasma samples from patients and healthy individuals, we used machine learning to identify eight key methylation signals from whole‑genome sequencing data. The diagnostic model we built, based on the Random Forest algorithm, performed remarkably well: it distinguished gastric cancer patients from healthy controls with high accuracy in an independent test set, achieving a sensitivity of over 94%. After adjusting for age, the model achieved an AUC of 0.924 on the test set, demonstrating high discriminatory performance. Importantly, the eight methylation markers we identified were overlooked by traditional epigenomic association studies based on microarrays (450K and EPIC v2). This was possible because we employed a technology that captures the entire methylation landscape. Our findings suggest that a simple blood test combined with our machine learning model has the potential to serve as a practical and noninvasive method for screening for gastric cancer. We believe this work brings us one step closer to making precision cancer detection more accessible and less burdensome for patients.

## Introduction

Gastric cancer (GC) remains a major global public health challenge, characterized by high incidence and mortality and ranked fourth worldwide, with a particularly heavy burden in East Asian countries such as China [1]. In 2020 alone, China reported approximately 479,000 new GC cases and 374,000 deaths, making it the third most common cancer in the country [2]. Although treatment options have expanded to include radical resection, chemotherapy, radiotherapy, targeted therapy, and immunotherapy [3], the prognosis for patients with advanced or metastatic disease remains poor. The 5-year overall survival rate in such cases is typically below 30%, significantly lower than the approximately 95% rate observed in early-stage GC [4,5]. Due to the nonspecific and insidious nature of early symptoms, the majority of patients are diagnosed at an advanced stage with limited therapeutic options [3], timely diagnosis and treatment help improve patient survival rates.

Currently, gastroscopy serves as the gold standard for GC diagnosis. However, its invasiveness, high cost and dependence on operator skill restrict its utility as a large-scale screening tool [6].Furthermore, conventional serum tumor markers (e.g., CEA and CA19–9) exhibit suboptimal sensitivity and specificity [7]. Consequently, there is an urgent need for a non-invasive and more accurate method for detecting GC.

Liquid biopsy based on circulating cell-free DNA (cfDNA) has gained increasing attention in recent years as a minimally invasive and easily accessible diagnostic modality. cfDNA primarily originates from apoptosis, necrosis, and active secretion, and is widely present in various bodily fluids such as blood [8]. In cancer patients, cfDNA in bodily fluids carries tumor-specific genomic and epigenetic information, including methylation patterns, mutations, and copy number variations, offering new avenues for non-invasive diagnosis and prognostic assessment [9]. Among these biomarkers, DNA methylation represents a key epigenetic mechanism that regulates chromatin structure, gene expression, and genomic stability. DNA methylation undergoes stable alterations in the early stages of tumorigenesis, making it a promising biomarker for early cancer detection [10]. Notably, aberrant methylation signals can be detected in circulating cfDNA even during early-stage cancer or pre-malignant conditions [11–13], supporting the potential of blood-based cfDNA methylation analysis for gastric cancer diagnosis.

Whole-genome bisulfite sequencing (WGBS) is considered the gold standard for DNA methylation analysis, covering nearly all cytosines across the genome and providing a powerful tool for the discovery of novel biomarkers [14]. Previous studies have utilized reduced-representation bisulfite sequencing (RRBS) or methylation chip technologies to identify methylation markers in GC tissues, while others have employed plasma samples to identify GC-specific methylation diagnostic markers [5]. However, these detection methods suffer from limitations such as low resolution and poor genomic coverage. Additionally, plasma cfDNA is derived from a mixture of cellular sources, not all of which are tumor-related [8]. As a result, studies relying solely on a single sample type may struggle to identify methylation changes that are both cancer-specific and reliably detectable in plasma.

Therefore, this study applied WGBS within a rigorous “tissue-to-plasma” translational framework—discovering methylation markers in tissues and validating them in plasma—to identify tumor-derived methylation signals that remain stable in circulation. By integrating machine learning algorithms, we developed a highly accurate and interpretable diagnostic model based on plasma cfDNA methylation, providing a novel strategy for the non-invasive detection of GC (Fig 1).

**Fig 1:**
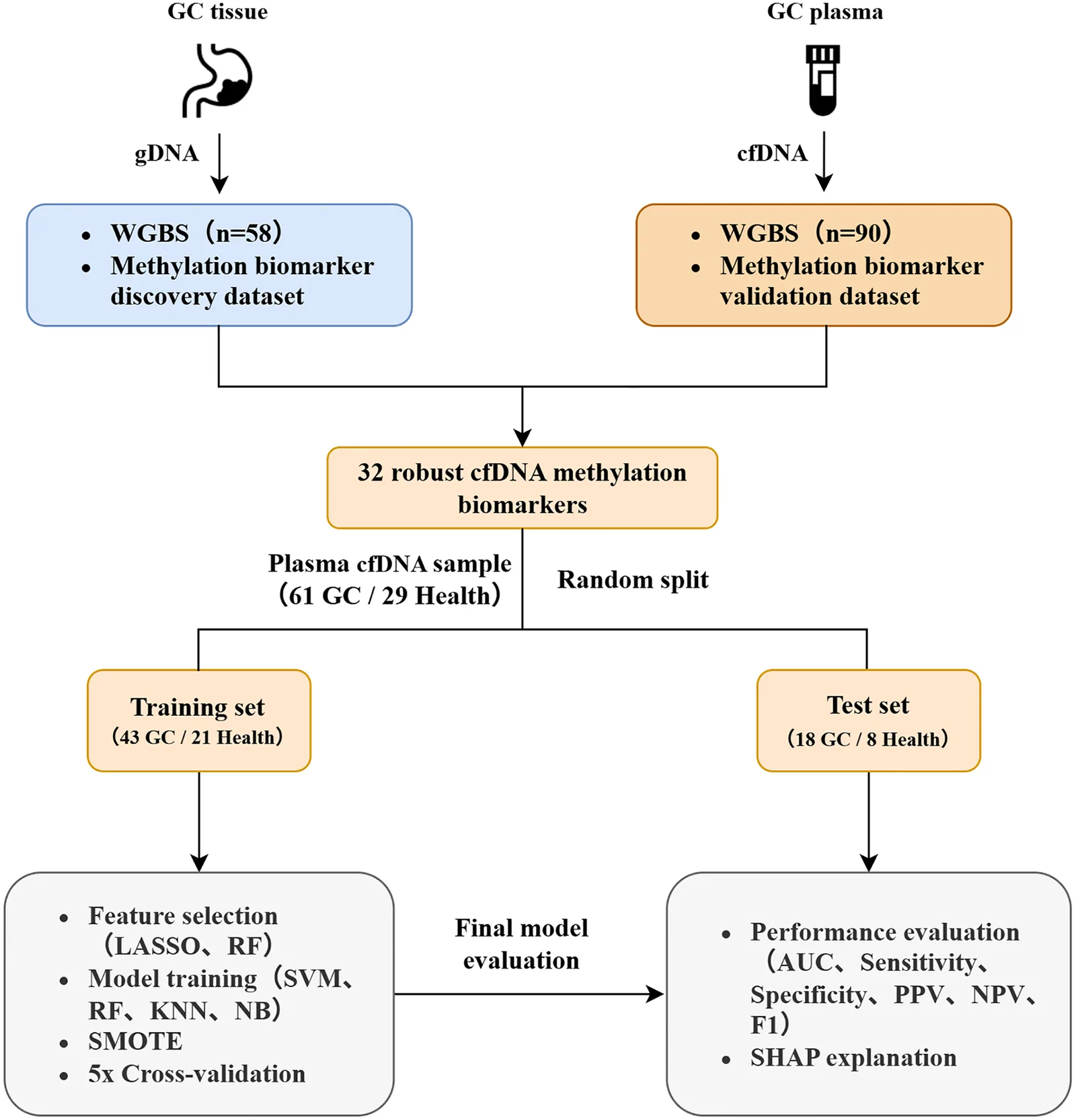
Schematic of the tissue-to-plasma discovery and validation workflow (Icons from the open-source website: https://healthicons.org/).

## Result

### Methylation profiles of tissue samples from GC patients

This study enrolled a total of 61 patients diagnosed with GC. WGBS was first performed on GC tissues and matched adjacent normal tissues from 29 of these patients. The cohort consisted predominantly of males (62.07%), with a mean age of 59.24 ± 13.81 years, and none of the patients reported a family history of GC. Clinical characteristics are summarized in S1 Table. Sequencing quality metrics show that the Q20 and Q30 hit rates exceeded 96% and 91%, respectively; the alignment rate for all samples was above 86%; and the disulfide conversion rate consistently remained above 99%, confirming the reliability of the estimated CpG methylation levels (S2 Table).

WGBS revealed significantly lower average methylation levels in the GC tissues than in adjacent normal controls (Fig 2A). Across functional genomic regions, normal tissues generally exhibited higher methylation levels than cancer tissues, except within CpG islands (CGIs), where the cancer group showed elevated methylation (Fig 2B). Using the Metilene software, a total of 260,038 DMRs were identified, of which 11.25% were hypermethylated regions (hyper) and 88.75% were hypomethylated regions (hypo), with an average length of 245 bp (Fig 2C). Enrichment analysis relative to the background regions showed that these DMRs are significantly enriched in regions such as promoters, the upstream 2 kb, CpG islands, transcription start sites (TSS)-associated CpG islands, and CpG shores; they are significantly depleted in regions such as the downstream 2 kb, introns, and gene body (Fig 2D). These DMRs were distributed across all chromosomes (Fig 2E).

**Fig 2:**
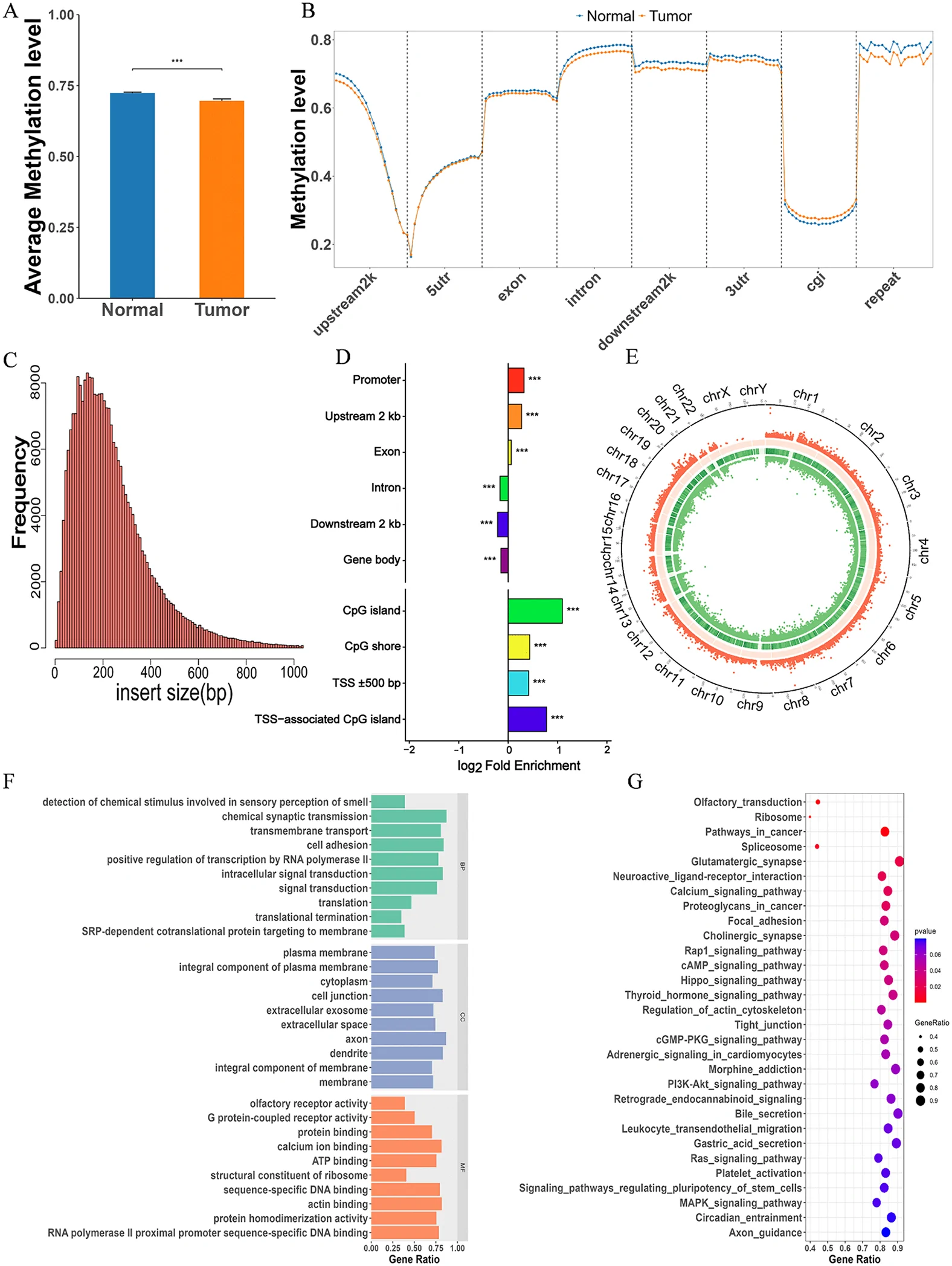
Methylation characteristics of gastric cancer tissues. (A) Overall methylation levels in gastric cancer tissues versus adjacent non-cancerous tissues; (B) Methylation levels of various genomic functional elements in gastric cancer tissue versus adjacent non-cancerous tissue; (C) Histogram of DMR length distribution; (D) The enrichment situation of DMR relative to the random control regions; (E) Circos plot showing the significance of DMR distribution across the entire genome; (F) GO enrichment results for DMR-associated genes; (G) KEGG enrichment results for DMR-associated genes.

To explore the functional significance of these DMRs, Gene Ontology (GO) and KEGG enrichment analyses were performed on DMR-associated genes (DMGs) based on genomic annotation. GO analysis indicated that these DMGs were primarily associated with cell adhesion, signal transduction, calcium ion binding, and other functions (Fig 2F). KEGG analysis revealed that these DMGs participated in multiple pathways associated with cancer development, including the Rap1 signaling pathway, cAMP signaling pathway, Hippo signaling pathway, PI3K-Akt signaling pathway, Ras signaling pathway, and cGMP-PKG signaling pathway (Fig 2G). These results suggest that methylation alterations may contribute to GC pathogenesis through modulation of these signaling cascades.

### Methylation profiles in plasma samples from GC patients

To identify non-invasive diagnostic biomarkers, we performed WGBS on plasma samples from all 61 GC patients and 29 healthy controls. Demographic characteristics are provided in S3 Table. The mean age was 59.38 ± 11.21 years in the cancer group and 55.48 ± 8.63 years in the healthy group, with no significant differences in age, sex, ethnicity, BMI, marital status, occupation, or family history of GC between groups.

Plasma methylation profiles closely mirrored those observed in tissues. The GC group also exhibited significantly lower average methylation levels than the healthy group (Fig 3A). Similarly, the GC group showed higher methylation levels in CGI regions than the healthy group (Fig 3B). A total of 3,491 differentially methylated regions (DMRs) were identified in plasma samples, comprising 76.05% hypomethylated regions and 23.95% hypermethylated regions, with an average length of 161 bp (Fig 3C). Enrichment analysis relative to the background region showed that these DMRs were significantly enriched in the promoter, upstream 2 kb, CpG island, TSS ± 500 bp, CpG shore, and exon regions (Fig 3D). These DMRs were distributed across all chromosomes (Fig 3E).

**Fig 3:**
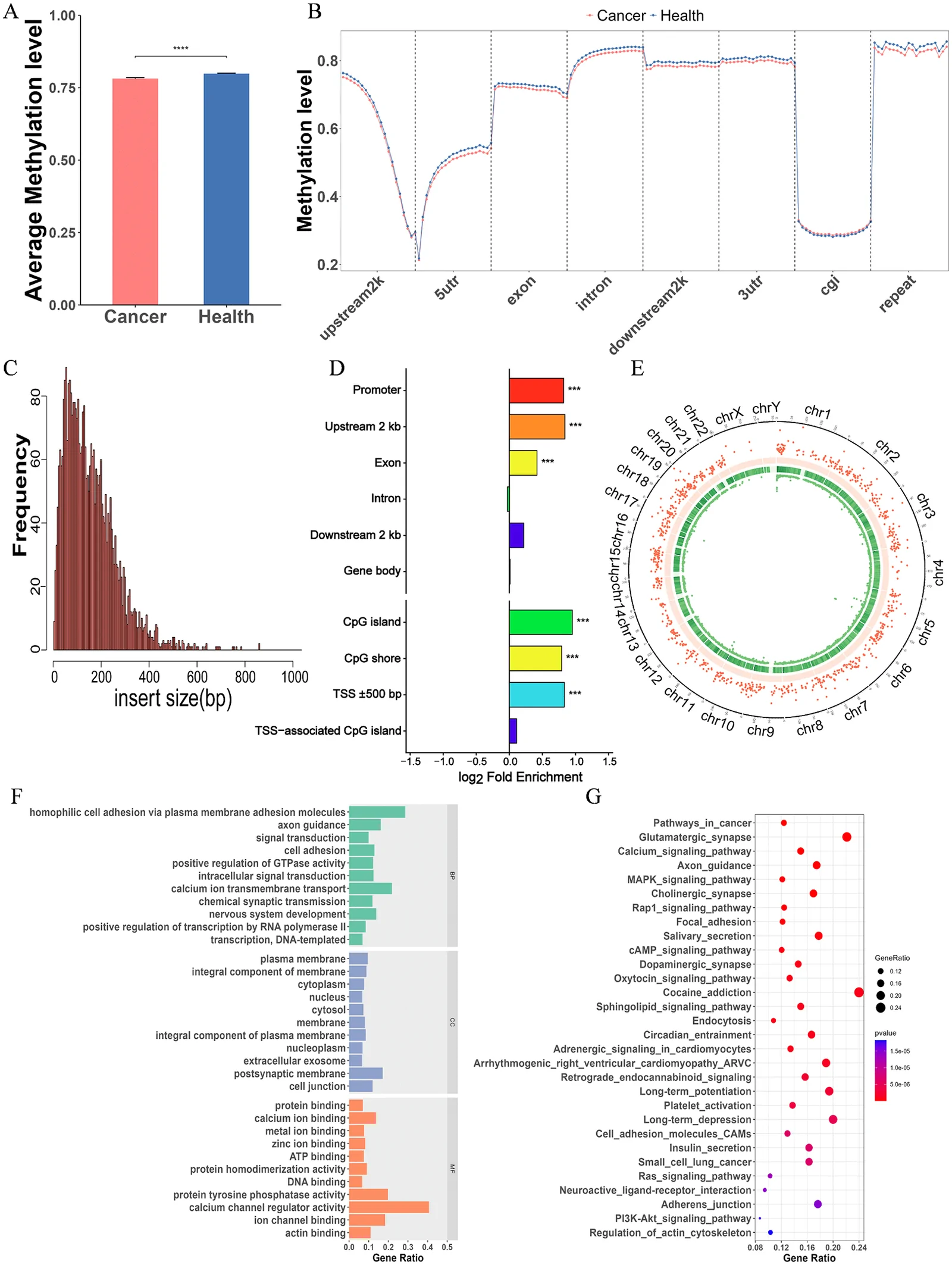
Methylation characteristics in gastric cancer plasma. (A) Overall methylation levels in gastric cancer plasma versus healthy plasma; (B) Methylation levels of genomic functional elements in gastric cancer plasma versus healthy plasma; (C) Histogram of DMR length distribution; (D) The enrichment situation of DMR relative to the random control regions; (E) Circos plot showing the significance of DMR distribution across the genome; (F) GO enrichment results for DMR-associated genes; (G) KEGG enrichment results for DMR-associated genes.

GO analysis of plasma-derived DMGs revealed enrichment in functions including calcium ion binding, signal transduction, cell adhesion, and ATP binding (Fig 3F). KEGG analysis revealed that these DMGs were enriched in pathways including calcium signaling, MAPK signaling, Rap1 signaling, cAMP signaling, Ras signaling, and PI3K-Akt signaling (Fig 3G). The similarity between plasma and tissue sample results indicates conserved methylation patterns across different sample types.

### Identification of plasma DMR biomarkers for non-invasive diagnosis

Methylation changes in plasma cfDNA may not fully reflect those present in primary tumors [15]. Our results also confirm this. The statistical analysis of the overlap between the DMRs from the tissue sources and the DMRs in plasma cfDNA shows that 15.12% (n = 528) of the plasma DMRs have at least 1 bp of genomic position overlap with the tissue DMRs. Additionally, 37 DMRs are common to both the tissue and plasma, accounting for 1.06% of the total plasma DMRs (**S3 Fig**). Among them, 2 DMRs have inconsistent methylation change directions in the tissue and plasma (**S4 Table**). Furthermore, at the functional pathway level, among the 131 KEGG pathways enriched by plasma DMRs, only 11 pathways (such as Pathways in Cancer, Calcium Signaling Pathway) are significantly enriched in the tissue as well (**S5** and **S6 Tables**).

In order to identify reliable epigenetic markers, we used DMRs that exhibited consistent changes in both tissue and plasma and were present in all plasma samples. A total of 32 such DMRs were selected for further analysis (**S7 Table**). A total of 26 patients in this study provided both tissue and blood samples. Individual-level correlation analysis of these samples revealed that 26 of the 32 consistent DMRs (81.25%) showed a positive correlation trend (**S8 Table**). Four DMRs reached statistical significance before correction (*p* < 0.05) but did not meet the significance threshold after correction for multiple comparisons (corrected *p* > 0.05), which may be related to the limited sample size of the paired samples (n = 26). Although statistical significance was limited, the overall trend supports that tissue-derived methylation changes can be partially reproduced in plasma cfDNA. Unsupervised clustering analysis revealed that these 32 DMRs were able to effectively distinguish GC patients from healthy individuals, as well as cancer tissues from adjacent tissues (Fig 4A and 4B), supporting their potential utility as diagnostic biomarkers.

**Fig 4:**
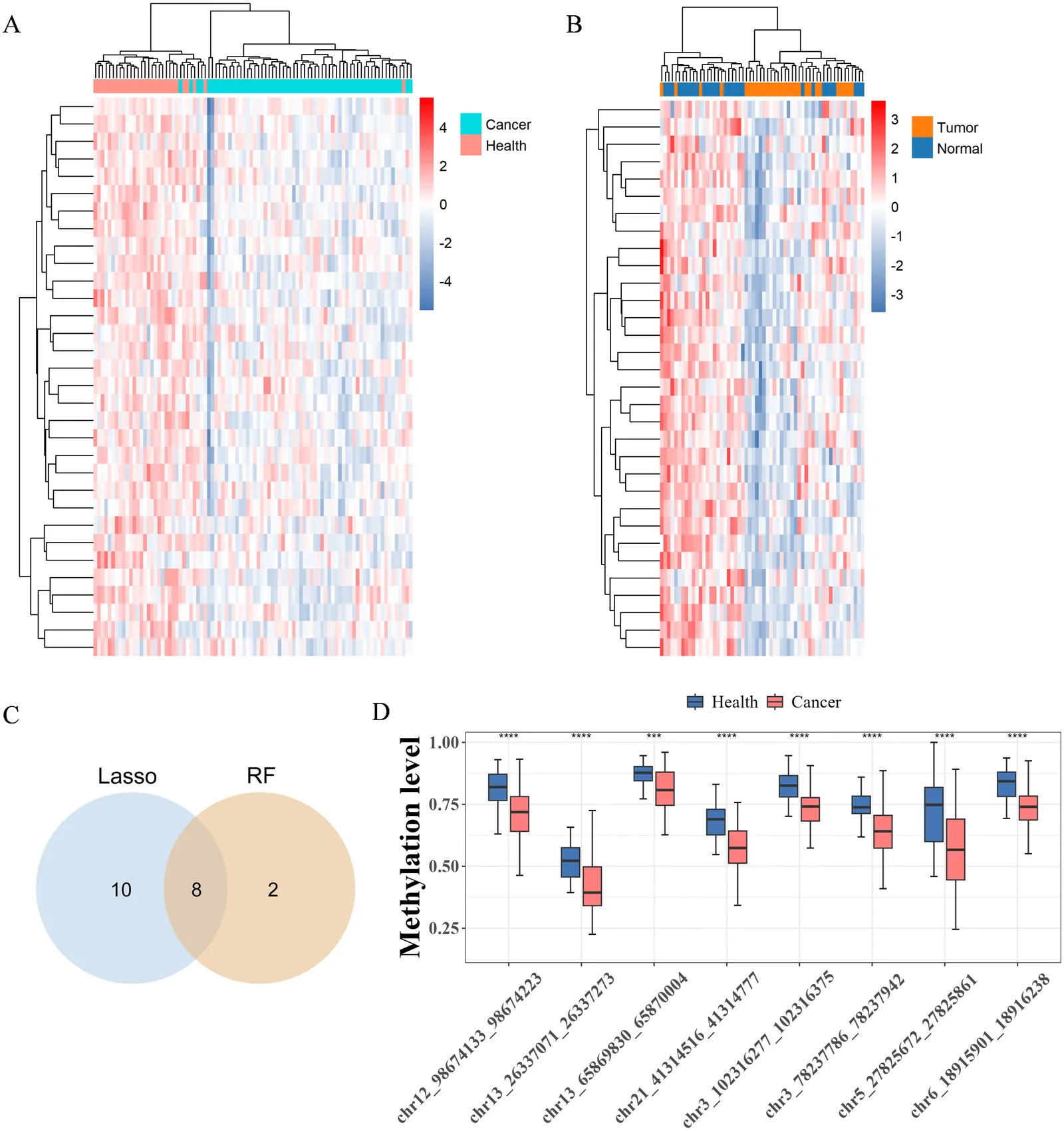
Identification of plasma DMR biomarkers. (A) Unsupervised clustering heatmap of 32 DMRs in plasma samples; (B) Unsupervised clustering heatmap of 32 DMRs in tissue samples; (C) DMRs overlapping between Lasso and RF screening methods; (D) Comparison of methylation levels for 8 DMRs in plasma samples from gastric cancer patients and healthy individuals.

To minimize redundant features and reduce the risk of overfitting, further feature selection was performed using both Least Absolute Shrinkage and Selection Operator (LASSO) and Random Forest (RF) methods. LASSO regression identified 18 non-zero coefficients (S1A and **S1B Fig**), while RF yielded the top 10 most important features (**S1C Fig**). This process ultimately identified 8 core cfDNA methylation diagnostic markers (Fig 4C). All 8 features exhibited a hypomethylation pattern in GC patients (Fig 4D).

### DMR-based methylation prediction model for GC

We constructed diagnostic models based on these eight DMRs using four machine learning algorithms in the training set. All models showed high discriminative performance, with AUC values exceeding 0.9 (Fig 5A). Specifically, the Support Vector Machine (SVM) model achieved an AUC of 0.957 (95% CI: 0.947–0.967), the RF model reached 0.978 (95% CI: 0.969–0.987), the K-Nearest Neighbors (KNN) model attained 0.963 (95% CI: 0.949–0.977), while the Naïve Bayes (NB) model achieved an AUC of 0.961 (95% CI: 0.953–0.969).

**Fig 5:**
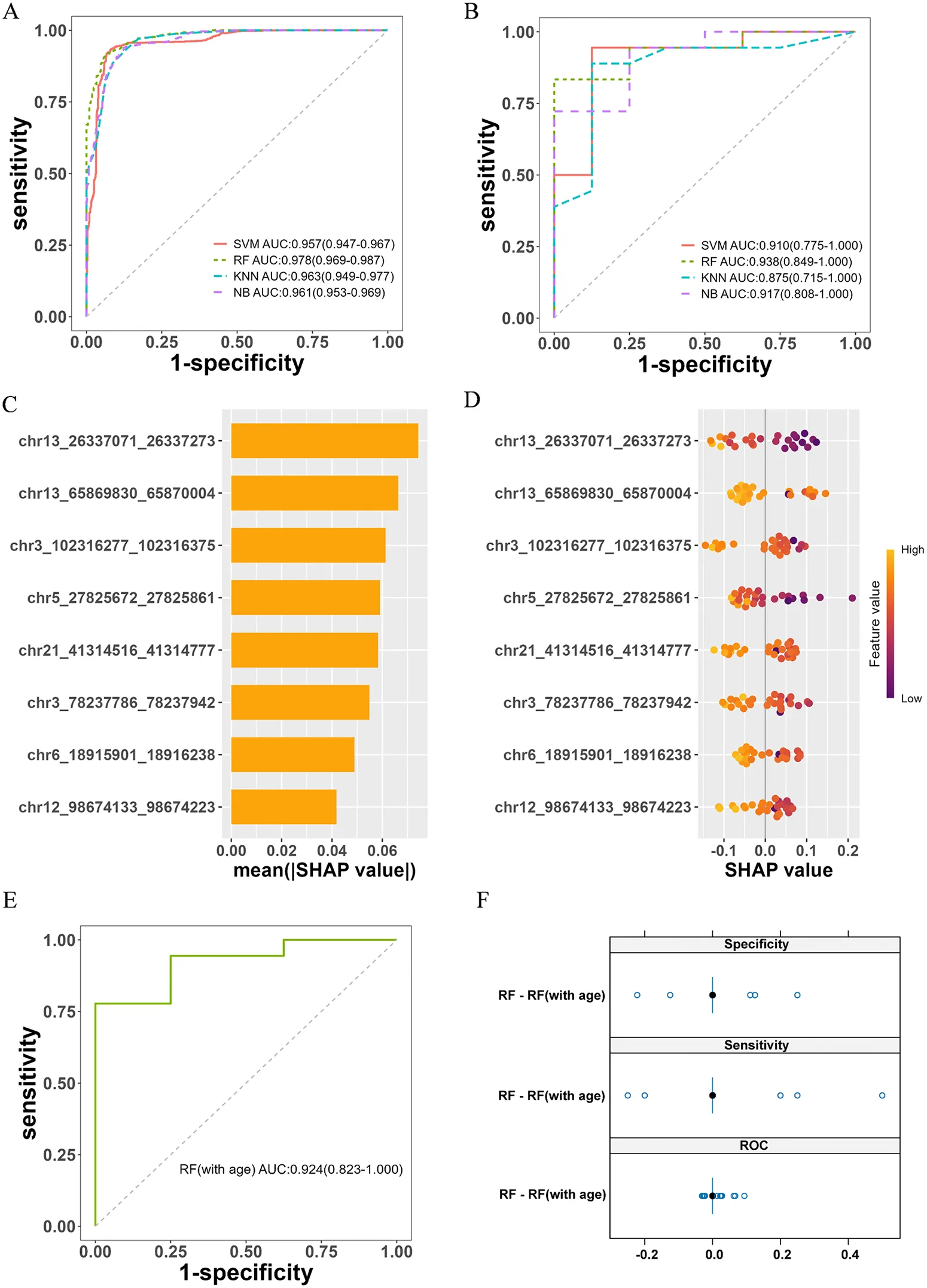
Gastric cancer prediction model based on cfDNA methylation biomarkers. (A) ROC curves of four machine learning models on the training set; (B) ROC curves of four machine learning models on the test set; (C) SHAP feature importance plot for the RF model; (D) SHAP explanation swarm plot for the RF model; data points represent samples, with color indicating the original value of the feature (more orange indicates a higher original value). The x-coordinate (SHAP value) of each point reflects the magnitude and direction of the feature’s influence on model prediction; (E) ROC curves of the age-corrected RF model on the test set;(F) Distribution of Performance Metrics Differences Among Different Models Based on Cross-Validation.

When evaluated on the test set, model performance remained robust, though a modest decrease in performance was observed (Fig 5B and Table 1). Among these, the RF model still demonstrated the best diagnostic performance with an AUC of 0.938 (95% CI: 0.849–1.000) and an accuracy of 88%. The SVM model achieved an AUC of 0.910 (95% CI: 0.775–1.000), the KNN model an AUC of 0.875 (95% CI: 0.715–1.000), and the NB model an AUC of 0.917 (95% CI: 0.808–1.000). Based on these results, the RF model was selected as the optimal classifier for GC diagnosis.

**Table 1:** Performance metrics of the models on the test set.

Models	Accuracy	Sensitivity	Specificity	PPV	NPV	F1
SVM	0.769	0.722	0.875	0.929	0.583	0.813
RF	0.885	0.944	0.750	0.895	0.857	0.919
KNN	0.885	0.889	0.875	0.941	0.778	0.914
NB	0.846	0.944	0.625	0.850	0.833	0.895
RF (with age)	0.846	0.889	0.750	0.889	0.750	0.889

Note: PPV = Positive predictive value, NPV = Negative predictive value

Due to the limited interpretability of machine learning models, we employed the SHAP method to interpret the top-performing RF model, enhancing understanding of its decision-making process. The SHAP feature importance plot displayed the SHAP value scores for these features (Fig 5C), with chr3_26337071_26337273 contributing most significantly to the model. The SHAP ensemble plot illustrated the decision-making roles of each feature in model predictions (Fig 5D), indicating that low methylation levels at these loci were associated with an increased probability of GC classification.

Since DNA methylation is age-related, to assess the potential confounding effect of age on the diagnostic model, we included age as a covariate in the optimal model (RF model) to control its impact on the classification effect. The results showed that after correcting for age, the AUC of the RF model in the independent test set was 0.924 (Fig 5E), and the accuracy rate was 84.62% (Table 1). Compared with before correcting age, the difference in the AUC value of the model after correcting age in the test set was not statistically significant (DeLong test, *p* = 0.403). Further statistical comparison based on 5-fold 10-repeated cross-validation showed that the differences in ROC, sensitivity, and specificity of the model after correcting age were all not statistically significant (P values were 0.259, 0.860, and 0.069, respectively) (Fig 5F). These results indicate that after correcting age, our main findings remain robust.

### Functional characterization and expression verification of methylation markers

Based on the excellent efficacy of the 8 methylation markers selected in the previous screening for the differential diagnosis of GC, this part further conducts functional exploration and expression verification. Compared with the normal control group, the 8 loci showed low methylation status in all stages of GC; and the methylation levels of chr12_98674133_98674223, chr13_26337071_26337273, chr21_41314516_41314777, chr3_102316277_102316375, and chr5_27825672_27825861 at these loci gradually decreased with the progression of tumor stage (Fig 6A). Although this trend was not statistically significant, it suggests that the methylation status of these loci is potentially associated with the stage of GC.

**Fig 6:**
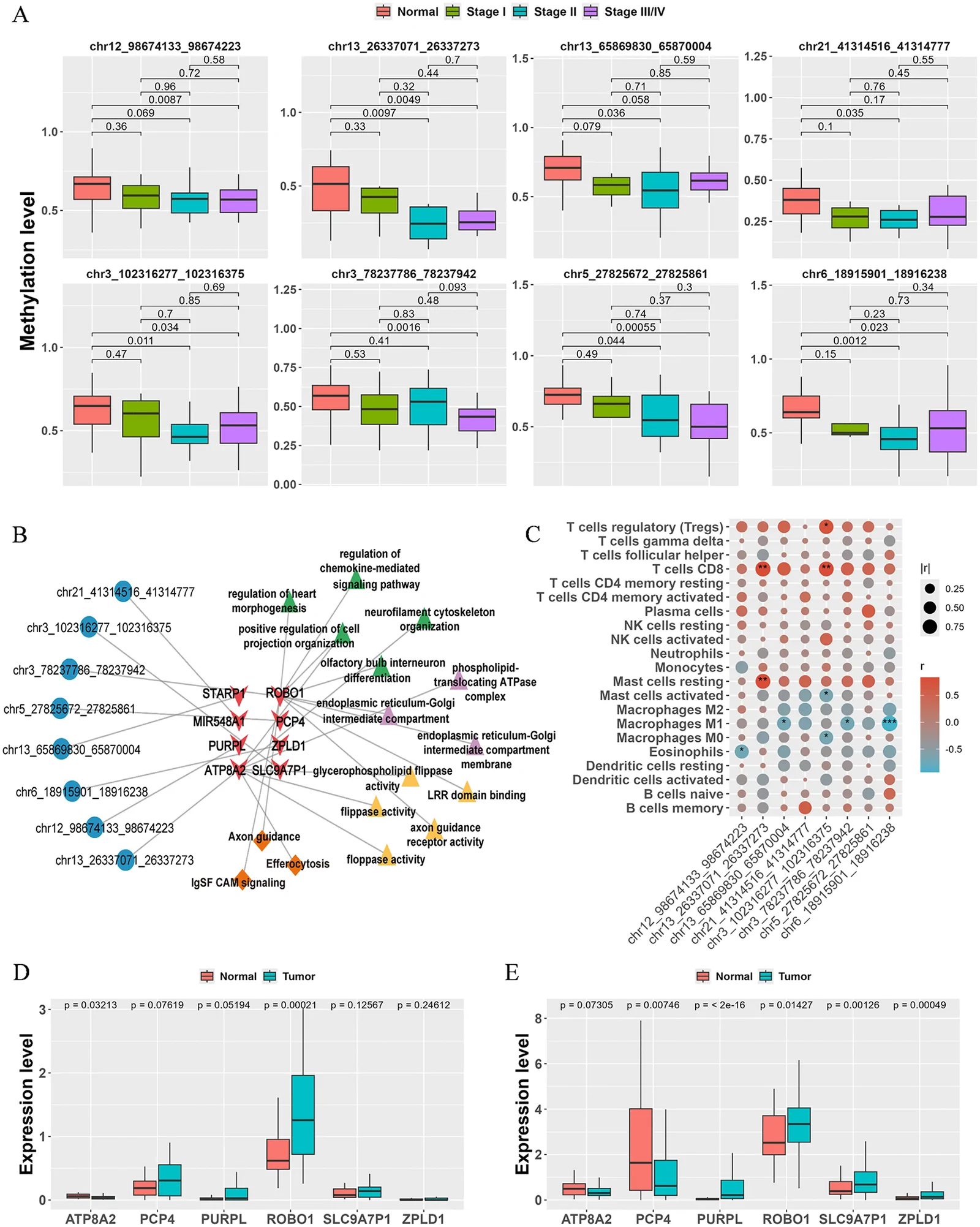
Function and expression of methylation markers. (A) Expression of 8 core DMRs at different stages; (B) DMR-gene-function network diagram, blue circles represent DMRs, red V-shapes represent genes, triangles represent GO analysis (green, purple, and yellow represent BP, CC, and MF modules respectively), orange rhombuses represent KEGG pathways; (C) Correlation analysis results of DMRs and immune infiltration; (D) The expression patterns of the DMR annotated genes in our transcriptome results across different samples (n = 57); (E) Expression of DMR annotated genes in different samples in the TCGA database. * indicates P < 0.05; ** indicates P < 0.01; *** indicates P < 0.001.

To clarify the target genes and biological functions of the core methylation markers, the unannotated loci were re-annotated at the genomic level and then GO and KEGG functional enrichment analyses were conducted for the corresponding target genes of the 8 loci. The GO enrichment results indicated that these genes were related to the positive regulation of cell protrusion, the regulation of chemokine-mediated signaling pathways, the phospholipid-transporting ATPase complex, the activity of axon guidance receptors, and the binding of LRR domains (Fig 6B). The KEGG enrichment results indicated that the target genes of these loci were involved in biological pathways such as phagocytosis, axon guidance, and IgSF cell adhesion molecule signal transduction (Fig 6B).

Previous studies have confirmed that phagocytosis plays an important role in regulating the cancer immune microenvironment and mediating cancer immune escape [16]. Based on this, the correlation between the methylation levels of the core loci and tumor immune cell infiltration was further analyzed. The immune infiltration correlation analysis showed that the methylation levels of each core locus were specifically associated with the abundance of specific immune cell subsets: chr12_98674133_98674223 was negatively correlated with eosinophils, chr13_26337071_26337273 was positively correlated with CD8 + T cells and resting mast cells, chr13_65869830_65870004 was negatively correlated with M1-type macrophages, chr3_102316277_102316375 was negatively correlated with regulatory T cells (Tregs) and CD8 + T cells, activated mast cells and M0-type macrophages, whereas chr3_78237786_78237942 and chr6_18915901_18916238 were negatively correlated with M1-type macrophages (Fig 6C). These results suggest that the abnormal low methylation of the core loci may participate in the occurrence and development of GC by regulating the infiltration pattern of tumor immune cells.

DNA methylation is a key epigenetic mechanism regulating gene transcription. Therefore, transcriptomic analyse were further performed for genes annotated to the identified methylation markers. The results demonstrated that ATP8A2 expression was downregulated in GC compared with normal tissues, where PCP4, ZPLD1, ROBO1, SLC9A7P1, and STARP1 exhibited trends toward increased expression (Fig 6D). We further utilized patient samples with both methylation and transcriptome data (n = 11) to conduct an exploratory analysis of the methylation-expression correlation for 6 biomarkers with expression data. The results (S9 Table) showed that the methylation levels of 3 genes (ROBO1, PCP4, SLC9A7P1) were negatively correlated with expression, while 3 genes (ATP8A2, PURPL, ZPLD1) showed a positive correlation. This may be due to the insufficient sample size of paired samples, resulting in insufficient test power and failing to reach statistical significance (*p* > 0.05). This result indicates that under the sample size of this study, no consistent linear regulatory pattern of methylation-gene expression was observed. Subsequently, we further verified the expression of these genes using the TCGA public database. Due to the methodological differences between the Illumina 450K/EPIC methylation chips used by the TCGA platform and our methylation data, it was not possible to directly verify the core DMRs discovered in this study. Only the expression of the corresponding annotated genes was explored. In the TCGA database, except for PCP4, the remaining genes exhibited expression patterns consistent with our sequencing data (Fig 6E). Although TCGA cannot verify the methylation sites themselves, it provides strong external support for the transcriptional dysregulation trend of these 6 genes in gastric cancer. These results provide preliminary clues for the functional exploration of the biomarkers and need to be further verified in larger samples.

## Discussion

This study first identified DMRsbetween GC tissues and paired adjacent normal tissues using WGBS to characterize GC-specific somatic methylation alterations. Subsequently, tissue-derived DMRs were mapped to plasma cfDNA methylation data, yielding 32 robust DMR biomarkers detectable in blood. Through feature selection using LASSO regression and RF algorithms, eight optimal cfDNA methylation diagnostic biomarkers were identified. A machine learning diagnostic model based on these eight loci demonstrated robust discriminatory performance, achieving AUC values of 0.978 and 0.938 in the training and independent validation sets, respectively. At a classification threshold of 0.5, the model attained an accuracy of 88.46%, with high sensitivity (94.44%) and moderate specificity (75.00%), effectively distinguishing GC patients from healthy individuals. After adjusting for age, the model achieved an AUC of 0.924 on the test set, still demonstrating high discriminatory performance. SHAP analysis elucidated the contributions of these epigenetic features, facilitating the identification of key biomarkers and their potential roles in GC pathogenesis.

cfDNA harbors genetic signatures originating from tumor cells, rendering blood-based DNA methylation increasingly valuable for diagnostic applications [17]. Clinical cfDNA methylation-based diagnostic kits are already available for cancers such as hepatocellular carcinoma, colorectal cancer, lung cancer, and prostate cancer [11]. Prior studies utilizing RRBS [18], 450K methylation arrays [19], or cfMeDIP-seq [20] have reported promising diagnostic biomarkers for GC. However, the short fragment length and low concentration of cfDNA in plasma necessitate deep and accurate sequencing for reliable assessment of genetic variants [21,22].RRBS interrogates only a subset of genomic CpG sites (approximately 6–12%), predominantly within promoter regions, potentially overlooking important methylation markers located in gene bodies, introns, or intergenic regions [23]. Methylation arrays (450K/EPIC) offer broader coverage than RRBS but rely on predefined probes, limiting the discovery of novel biomarkers [24]. Enrichment-based methods (MeDIP-seq/cfMeDIP-seq) favor low-density methylation regions and also suffer from limited resolution and genome coverage [25,26]. In contrast, WGBS provides single-base resolution and covers over 90% of CpG sites across the genome, remaining the gold standard for comprehensive, unbiased DNA methylation analysis [23,26]. Our findings support these advantages. First, we identified over 260,000 tissue DMRs distributed across gene bodies, introns, and intergenic regions (Fig 2D). Notably, our overlap analysis of the 32 concordant DMRs revealed that only one non-core DMR (genomic region chr1:208619381–208619490) showed partial read coverage in the EPIC v2 methylation array and RRBS dataset (GSE55159) (S10 Table). The eight key DMRs used in the diagnostic model were not covered by physical probes on either the 450K or EPIC v2 methylation chips(S10 Table and S2 Fig), indicating their unique value as novel biomarkers; these core diagnostic markers have been overlooked in traditional microarray-based epigenetic association studies. Therefore, our WGBS-based screening strategy not only validated regions implicated in previous studies but also expanded exploration into previously underexplored genomic regions, providing a robust technical foundation for identifying this novel plasma cfDNA methylation biomarker panel.

Whole-genome hypomethylation accompanied by locus-specific hypermethylation is a hallmark of most cancer genomes [27]. Global hypomethylation in cancer can induce chromatin rearrangement and decondensation, thereby promoting genomic instability and oncogenic mutations [28]. CGI regions, characterized by high CpG density and elevated G + C content, typically coincide with transcription start sites and are frequently hypermethylated in cancer cells [29,30]. Consistent with previous studies, GC tissues exhibited significantly lower overall methylation levels than normal tissues, whereas CGI regions showed higher methylation levels (Fig 2). DNA methylation patterns also demonstrate a high degree of conservation between tissue and plasma samples [31]. Our results confirm similar methylation profiles in both compartments, consistent with previous studies reporting conserved methylation patterns between tissue and plasma [31–33]. These findings support the utility of plasma DNA methylation as a viable liquid biopsy biomarker.

The high background noise inherent in plasma cfDNA—which does not originate exclusively from tumor cells—poses challenges for accurate cancer diagnosis [22]. To identify reliable methylation markers in plasma, we mapped tissue-derived DMRs to plasma cfDNA methylation data using adopting a “discovery in tissue, validation in plasma” workflow to minimize bias. Thirty-two robust DMRs were identified and subsequently refined to eight high-performance diagnostic markers. Notably, DNA methylation alterations in tumor tissues do not fully correspond to plasma cfDNA patterns [15]. Our data highlight this discrepancy: among the initial 37 overlapping methylation loci between tissue and plasma, two DMRs exhibited inconsistent methylation states (**S4 Table**). Consequently, studies based on a single sample type may inadequately capture tumor-specific methylation signals. Our integrated approach therefore improves the identification of high-value epigenetic markers. Previous biomarker studies have predominantly relied on a single sample type. For example, Anderson et al. [18] identified a panel of methylation markers in gastric adenocarcinoma tissues, with a three-marker combination detecting 86% of cases in plasma samples, slightly lower than the detection accuracy achieved in our study (88%). Kandimalla et al.[19] developed the 50-DMR EpiPanGIDx plasma diagnostic panel based on differential CpGs from six gastrointestinal cancers, achieving a median AUC of 0.87 for GC detection, whereas our AUC reached 0.938. In a recent blood-based study, 21 DMRs achieved high sensitivity and specificity (88.38% and 94.23%, respectively) [20]. However, the relatively large number of markers may hinder clinical feasibility. In contrast, our model achieved 94.44% sensitivity using only eight loci, suggesting higher screening efficiency and a more parsimonious model with stronger translational potential. This concise eight-loci panel may facilitate the future development of qPCR or multiplex PCR-based clinical assays with low cost, shorter turnaround time, and easier implementation in clinical laboratories.

We identified 32 robust DMR markers annotated to 13 genes (11 protein-coding genes, 2 non-coding genes; **S7 Table**), several of which have previously been implicated in cancer development [34–41]. The eight core DMRs were annotated primarily to two genes ATP8A2 and STARP1. Among these, ATP8A2 has been more extensively studied in cancer contexts. ATP8A2, a nuclear mitochondrial gene [42], encodes ATPase phospholipid transporter 8A2, a member of the P4-ATPase family that actively translocates phosphatidylserine and phosphatidylethanolamine from the extracellular to cytoplasmic leaflet of the plasma membrane, thereby maintaining phospholipid asymmetry [43]. Due to its involvement in neurite elongation and neuronal survival, various ATP8A2 genomic variants have been identified in patients with neurological disorders [44]. To date, only limited studies have reported aberrant ATP8A2 expression in lung adenocarcinoma [45], early-onset intrahepatic cholangiocarcinoma [46], colon adenocarcinoma [47], and GC [48]. Previous studies demonstrated negative correlations between DNA methylation and ATP8A2 mRNA expression in several cancers, including lung, gastric, renal, breast, and liver cancers [49]. Although promoter methylation generally associated with transcriptional silencing of gene expression, increasing evidence suggests that gene body methylation may be positively correlated with gene expression [50]. Our results also observed this phenomenon. The methylation sites in the gene body region of ATP8A2 selected in this study were found to be hypomethylated in gastric cancer tissues, accompanied by a downregulation of expression (S4 Fig). Although the methylation-expression association analysis did not show statistical significance (S9 Table), the positive correlation trend suggested that the relationship between abnormal methylation and transcriptional regulation was location-specific. Interestingly, ATP8A2 silencing via hypermethylation has been reported in lung adenocarcinoma and several other cancers [42,51], suggesting potential tissue-specific epigenetic regulation. Although the role of ATP8A2 in GC remains poorly understood, a recent study identified ATP8A2 as a prognostic risk factor (HR > 1) in GC [48], further supporting its involvement in GC pathogenesis. Nevertheless, the potential tumor-suppressive role of ATP8A2 in GC requires further experimental validation. In addition, the biological fuctions of other marker-associated genes remain to be clarified.

To further investigate the biological relevance of the eight core DMRs, previously unannotated loci were reannotated, followed by functional enrichment analysis. The results demonstrated that the target genes were associated with biological pathways including phagocytosis, axon guidance, and IgSF cell adhesion molecule signaling. Phagocytosis plays an important role in regulating the tumor immune microenvironment and mediating immune evasion [16]. Therefore, we further analyzed correlations between methylation levels and immune cell infiltration. Several loci exhibited significant associations with specific immune cell subsets (Fig 6C). CD8 + T cells, which are critical cytotoxic T lymphocytes involved in antitumor immunity, are known to undergo exhaustion in GC [52]. Our results demonstrated that chr13_26337071_26337273 and chr3_102316277_102316375 were positively correlated with CD8 + T cell abundance. Previous studies have also shown that ATP8A2 expression is downregulated in GC and positively associated with immune and stromal cells infiltrations, including CD8 + T cells [48]. These findings further suggest that hypomethylation at this genomic region may influences ATP8A2 expression and subsequently affect CD8 + T cell infiltration with in the tumor microenvironment (TME). In addition, several target genes associated with other methylation markers have also been implicated in TME regulation. For example, genetic inhibition of ROBO1 signaling in glioblastoma reduces macrophage recruitment into the TME and prevents macrophage polarization toward a tumor-supportive phenotype [53]. In bladder cancer, high ZPLD1 expression is positively correlated with Th1 cells, macrophages, neutrophils, and NK cells [54]. Similarly, PURPL overexpression in lung cancer promotes M2 macrophage polarization [55]. However, given the complexity of TME, which includes tumor cells, immune cells, stromal cells, surrounding vasculature, and signaling molecules [56], the precise roles of these methylation markers in GC require additional investigation. Collectively, these findings suggest that methylation alterations may regulate target gene expression and thereby influence the immune microenvironment during gastric tumorigenesis.

Several limitations of this study should be acknowledgment. First, the study cohort was derived from a single center and had a relatively small size, and external validation cohorts were not available. Although internal validation performance was strong, the generalizability of the model requires confirmation in multicenter studies with large independent cohorts. Secondly, the small sample size of paired samples in this study resulted in insufficient statistical power, which limited our analysis in two ways: on the one hand, no consistent linear regulation pattern between methylation and gene expression was observed; on the other hand, although most DMRs showed a positive correlation trend between tissue and plasma, this trend did not reach statistical significance. It should be emphasized that epigenetics plays a crucial role in transcriptional regulation; therefore, the association between methylation and gene expression remains one of the key areas for further validation in this study. Furthermore, the consistency of these biomarkers at the individual level needs to be further validated in a larger-scale paired cohort to clarify their reliability for clinical applications. Third, because methylation alterations may also occur in benign gastric lesions [57], the absence of benign gastric disease cohorts (e.g., chronic gastritis or gastric ulcers) limits assessment of the model’s specificity for distinguishing GC from nonmalignant gastric conditions. Fourth, class imbalance existed in model-building dataset. Althoug SMOTE improved model training performance, the robustness of this approach requires validation in naturally balanced real-world populations. Finally, due to the lack of pre-diagnostic longitudinal samples, we believe that the purpose of this test is to provide a blood-based, noninvasive screening option for symptomatic patients, particularly those who are reluctant to undergo invasive gastroscopy. The sample size in the early phase of this study (Phase I) was small, and its ability to screen for early-stage disease still needs to be validated in a larger, independent cohort.

In summary, using WGBS and a “tissue-plasma” concordance analysis strategy, we identified a robust cfDNA methylation biomarker panel for GC detection. The machine learning diagnostic model constructed from eight methylation markers demonstrated strong discriminatory performance, supporting the potential clinical utility of plasma cfDNA methylation for noninvasive GC screening.

## Conclusion

This study employed gold-standard WGBS detection technology and a “tissue-plasma” concordance analysis strategy to identify eight robust plasma cfDNA methylation biomarkers. The machine learning diagnostic model constructed using these biomarkers demonstrated strong discriminatory performance in distinguishing GC patients from healthy individuals, highlighting the potential of plasma cfDNA methylation analysis as a non-invasive strategy of GC screening.

## Materials and methods

### Study population and sample collection

This cohort study enrolled 61 GC patients and 29 healthy controls. From each GC patient, paired biopsy tissue (pathologically confirmed tumor tissue) and plasma samples were collected, while only plasma samples were obtained from the healthy controls. All participants were recruited from the Affiliated Cancer Hospital of Guangxi Medical University and provided written informed consent (Ethics Approval Number: KY2023749). All fresh samples were snap-frozen immediately after collection and stored at -80 °C. All procedures were conducted in accordance with protocols approved by the Institutional Review Board and conformed to the principles of the Declaration of Helsinki. Patient medical records and pathology reports were reviewed by experienced hospital pathologists. Detailed clinical characteristics and sample information used in sequencing analyses are summarized in **S1**, S3 and S11 Tables.

### Sample preparation and DNA extraction

Plasma Sample Collection and Preservation: Blood samples were collected using EDTA-containing anticoagulant tubes. Plasma was immediately separated and aliquoted. Plasma samples were rapidly frozen in liquid nitrogen and stored at -80°C. cfDNA was extracted from approximately 10 mL of plasma using a cfDNA isolation kit.

Tissue Sample Collection and Preservation: GC tissue and adjacent normal tissue were obtained from GC patients. Tissue was sectioned into soybean-sized pieces and aliquoted into tubes. Tissue samples were immediately cryopreserved in liquid nitrogen and stored at -80°C. DNA was extracted from the tissue using a DNA extraction kit.

### Whole-genome bisulfite sequencing and data analysis

Among the 61 patients with GC, paired tumor and adjacent normal tissues from 29 patients were subjected to WGBS for tissue methylation profiling, whereas plasma cfDNA samples from all 61 patients and 29 healthy controls were included in the plasma methylation analysis. After sample DNA passed quality control, at least 0.5 μg of genomic DNA was extracted. The DNA was mixed with 1‰ unmodified lambda DNA and 1‰ PUC19 DNA methylated at CG sites. The mixture was ultrasonically fragmented to a primary band size range of 200–500 bp, then treated with oxidation and DEA enzymes. Subsequently, the DNA was denatured into single strands and add 3’-adapters and tails were added. Subsequently, the 3’-adapter-ligated single-stranded DNA was extended into double stranded DNAs, followed by 5’-adapter ligation. The ligation products underwent approximately 10 cycles of PCR amplification using double-ended 8-bp tag primers. The library was quality-assessed using an Agilent 2100 system, and final selection was made for double-ended 150-bp sequencing. Raw data underwent quality assessment using FastQC software, followed by Trimmomatic software to trim sequencing adapters and low-quality data. The resulting clean data were used for subsequent analysis. BSMAP software was employed to map methylation data against the reference genome (hg19), simultaneously identifying methylation sites, analyzing methylation sequence types, and calculating site-specific methylation levels. Differentially methylated regions (DMRs) were identified using the metilene software. Regions with a corrected p-value < 0.05 and an absolute difference in methylation level > 0.10 were defined as DMRs. For unmapped DMRs, the ChIPseeker software package was used to re-annotate based on the coordinates of the methylation regions. The ChIPseeker package was used to calculate the relative positional relationship between the given genomic intervals and the known genomic features (genes, exons, promoters, etc.). The genomic annotation file was based on the hg19 version, and the promoter range is defined as ±2000 bp.

### Enrichment assay for DMRs

For each true DMR, a genomic region of exactly the same length was randomly selected from the same chromosome, excluding regions that overlapped with the true DMR or contained ambiguous bases from the reference genome. A total of 10,000 sets of random control regions were generated based on the distribution of the number and length of true DMRs. Both the true DMRs and the random control regions were annotated using the same hg19 functional element annotation file and the same overlap criteria. The enrichment fold was calculated as the ratio of the proportion of true DMRs within each functional element to the average proportion in the random background. P-values were calculated using a two-sided empirical permutation test, followed by Benjamini–Hochberg correction for multiple comparisons.

### Transcriptome sequencing

Total RNA was extracted using TRIzol reagent. The quantity and purity of the total RNA were quality-controlled, and the integrity of the RNA was detected using the Bioanalyzer 2100. mRNA with PolyA (polyadenylic acid) was specifically captured using oligo(dT) magnetic beads through two rounds of purification. The captured mRNA was fragmented at high temperature using the magnesium ion fragmentation kit. The fragmented RNA was synthesized into cDNA by the action of reverse transcriptase. Then, fragments of size 300 bp ± 50 bp were formed by PCR. The original data was sequenced using Illumina Novaseq 6000 according to the standard operation, with a sequencing mode of PE150. Cutadapt was used to further filter the raw data, and the effective data was obtained for subsequent analysis. Hisat2 was used for reference genome alignment. Based on the alignment results of Hisat2, StringTie was used to reconstruct transcripts and calculate the expression levels of all genes in each sample. The expression abundance of mRNA was determined by calculating the FPKM (transcripts per million mapped reads per 1,000 base pairs) value. DESeq2 software was used to perform gene differential expression analysis on two different groups. Genes with a false discovery rate (FDR) parameter lower than 0.05 and an absolute fold change of ≥ 2 were considered differentially expressed genes.

### Feature selection and model building

Using the R package caret (v6.0.94), samples were randomly split into training and testing sets at a 7:3 ratio. The training set was used for model development, while the testing set served solely for model performance evaluation. There were no statistically significant differences in demographic and clinical characteristics between the training and test sets (*p* > 0.05) (S12 Table), indicating that our data were randomly and evenly stratified. To ensure the genomic regions considered by the model were covered across all samples, any rows with missing values in the methylation matrix were removed from the matrix. Subsequently, to identify the most important variables, reduce redundant features, and enhance model performance and interpretability, feature selection was performed using two methods: the Least Absolute Shrinkage and Selection Operator (LASSO) and Random Forest (RF). LASSO regression is a powerful regression analysis technique that employs L1 regularization, imposing a penalty proportional to the absolute value of coefficients. This effectively reduces the coefficients of less significant variables to zero, facilitating feature selection within the model. Random Forest is a widely used machine learning algorithm for classification, regression, and other tasks, serving as an effective method for selecting important variables from high-dimensional data. Simultaneously, the selected variables underwent variance inflation factor (VIF) testing to avoid multicollinearity among variables. Furthermore, our model dataset comprises 29 healthy individuals and 61 GC patients, exhibiting significant class imbalance. Without mitigation, the model would favor the majority class, adversely affecting machine learning algorithm performance. To address this, we employed the Synthetic Minority Over-sampling Technique (SMOTE) for class imbalance correction (K-nearest neighbors = 5). **S13 Table** reports model performance comparisons before and after SMOTE implementation. The R package caret provides a unified interface for implementing various machine learning algorithms, simplifying training and evaluation. We employed four machine learning algorithms for model construction: Support Vector Machine (SVM), Random Forest (RF), K-Nearest Neighbors (KNN), and Naïve Bayes (NB). Hyperparameter tuning for each model was performed using the R package caret. To enhance model accuracy and minimize fitting error, each model was trained using repeated 5-fold cross-validation (10 repetitions). The best-tuned model was selected based on AUC as the evaluation metric. The optimal tuning parameters for each model are listed in **S14 Table**.

### Model evaluation

Predictions were made on a completely independent test set, with AUC serving as the primary metric for selecting the optimal predictive model. A confusion matrix was calculated using a classification threshold of 0.5 prediction probability, reporting the following model performance metrics: accuracy (ACC), sensitivity, specificity, positive predictive value (PPV), negative predictive value (NPV), and F1 score. Additionally, SHAP analysis was performed to improve model interpretability and enhance understanding of the model’s decision-making process.

### Immune infiltration analysis

The CIBERSORT R package (version 0.1.0) was used to estimate the proportion of 22 types of immune cell populations in each sample. CIBERSORT employs Monte Carlo sampling to estimate the p-value, thereby evaluating the deconvolution results of each sample. To ensure accurate results, we conducted 1000 permutations to improve the robustness and accuracy of immune cell proportions estimates. For samples with a p-value < 0.05, Spearman correlation analysis was used to assess the relationship between the abundance of immune cells and the methylation level.

### Verification using the TCGA database

Using the TCGAbiolinks package (version 2.30.4), gene expression data from the TCGA-STAD project (421 GC samples and 36 normal samples) were obtained, and the expression levels of the corresponding genes were evaluated.

### Statistical analysis

All statistical analyses were performed using R software (version 4.2.2). Unsupervised clustering and heatmap analysis were conducted using the pheatmap package (version 1.0.12) in R. Perform genomic overlap analysis using the GenomicRanges package (version 1.54.1) in R. Receiver operating characteristic (ROC) analysis was performed using the “roc” function in the R package pROC (version 1.18.5), generating area under the curve (AUC) and 95% confidence intervals (CI) to assess model performance. SHAP analysis of machine learning models was performed using the kernelshap (version 0.7) and shapviz (version 0.9.3) packages. Use the ‘diff.resamples’ method from the caret package in R to conduct a significance test for the differences in model performance. Unless otherwise specified, adjusted p-values < 0.05 were considered statistically significant.

### Ethical approval

This research has been performed in accordance with the Declaration of Helsinki and approved by Ethics Committee of Guangxi Medical University Cancer Hospital (NO. KY2023749).

## Supporting information

S1 Fig Results of feature selection methods using Lasso and RF algorithms. (A) Path diagram of coefficient changes for the Lasso algorithm; (B) Cross-validation plot for the Lasso algorithm; (C) Feature importance ranking results for the RF algorithm.
(TIF)

S2 Fig Query results for 8 core DMRs using the UCSC Genome Browser. (A)DMR ID: chr3_78237786_78237942;(B) DMR ID: chr3_102316277_102316375; (C)DMR ID: chr5_27825672_27825861; (D)DMR ID: chr6_18915901_18916238; (E)DMR ID: chr12_98674133_98674223; (F)DMR ID: chr13_26337071_26337273; (G)DMR ID: chr13_65869830_65870004; (F)DMR ID: chr21_41314516_41314777.
(TIF)

S3 Fig Number of DMRs Shared by Tissue and Plasma. (TIF)

S4 Fig Expression and methylation levels of the ATP8A2 gene in gastric cancer tissue samples. (A) Methylation levels of the ATP8A2 gene in gastric cancer tissue samples; (B) Expression levels of the ATP8A2 gene in gastric cancer tissue samples.
(TIF)

S1 Table Summary Table of Information for 29 Gastric Cancer Patients. (XLSX)

S2 Table Quality Control Results for Sequencing Samples. (XLSX)

S3 Table Statistical summary table of patient information (n = 90). (XLSX)

S4 Table DMRs overlapping tissue and plasma. (XLSX)

S5 Table KEGG Enrichment Results for DMRs in Tissue Samples. (XLSX)

S6 Table KEGG Enrichment Results for DMRs in Plasma Samples. (XLSX)

S7 Table Annotation information for 32 consistent DMRs in blood. (XLSX)

S8 Table Correlation Analysis of 32 DMRs in Tumor Tissue and Plasma Samples. (XLSX)

S9 Table The correlation analysis results of the core DMRs with the annotated genes. (XLSX)

S10 Table Overlap statistics for all 32 candidate DMRs. (XLSX)

S11 Table Number of samples for NGS sequencing. (XLSX)

S12 Table Summary Table of Patient Information for the Training and Test Sets. (XLSX)

S13 Table Performance Metrics of RF Models Before and After SMOTE Implementation. (XLSX)

S14 Table Model Parameters. (XLSX)
